# Possible Roles of Plant Sulfurtransferases in Detoxification of Cyanide, Reactive Oxygen Species, Selected Heavy Metals and Arsenate

**DOI:** 10.3390/molecules20011410

**Published:** 2015-01-14

**Authors:** Parvin Most, Jutta Papenbrock

**Affiliations:** 1 Institute of Botany, Leibniz University Hannover, Herrenhäuserstr. 2, Hannover D-30419, Germany; E-Mail: shanaj777@gmail.com; 2 Plant Breeding Division, Bangladesh Agricultural Research Institute, Joydebpur, Gazipur 1701, Bangladesh

**Keywords:** arsenate, arsenate reductase, cyanide, rhodanese, sulfurtransferase

## Abstract

Plants and animals have evolved various potential mechanisms to surmount the adverse effects of heavy metal toxicity. Plants possess low molecular weight compounds containing sulfhydryl groups (-SH) that actively react with toxic metals. For instance, glutathione (γ-Glu-Cys-Gly) is a sulfur-containing tripeptide thiol and a substrate of cysteine-rich phytochelatins (γ-Glu-Cys)_2–11_-Gly (PCs). Phytochelatins react with heavy metal ions by glutathione S-transferase in the cytosol and afterwards they are sequestered into the vacuole for degradation. Furthermore, heavy metals induce reactive oxygen species (ROS), which directly or indirectly influence metabolic processes. Reduced glutathione (GSH) attributes as an antioxidant and participates to control ROS during stress. Maintenance of the GSH/GSSG ratio is important for cellular redox balance, which is crucial for the survival of the plants. In this context, sulfurtransferases (Str), also called rhodaneses, comprise a group of enzymes widely distributed in all phyla, paving the way for the transfer of a sulfur atom from suitable sulfur donors to nucleophilic sulfur acceptors, at least* in vitro*. The best characterized* in vitro* reaction is the transfer of a sulfane sulfur atom from thiosulfate to cyanide, leading to the formation of sulfite and thiocyanate. Plants as well as other organisms have multi-protein families (MPF) of Str. Despite the presence of Str activities in many living organisms, their physiological role has not been clarified unambiguously. In mammals, these proteins are involved in the elimination of cyanide released from cyanogenic compounds. However, their ubiquity suggests additional physiological functions. Furthermore, it is speculated that a member of the Str family acts as arsenate reductase (AR) and is involved in arsenate detoxification. In summary, the role of Str in detoxification processes is still not well understood but seems to be a major function in the organism.

## 1. Introduction

**Scheme 1:**
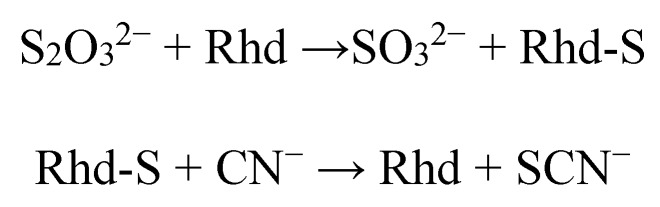
A sulfurtransferase reaction catalyzed by rhodanese.

Sulfurtransferases (Str), also called rhodaneses, catalyzes the transfer of a sulfur atom from suitable sulfur donors to nucleophilic sulfur acceptors [1]. The most studied and best characterized Str is bovine liver rhodanese that catalyzes* in vitro* the transfer of a sulfane sulfur atom from thiosulfate (TS) to cyanide, leading to the formation of sulfite and thiocyanate by forming a Rhod-S intermediate, which is characterized by a persulfide bond at the sulfhydryl group of the essential cysteine residue 247 (Scheme 1) [2]:

Rhodanese activity has been detected in all major phyla [3]. Str/rhodanese domains can be found as tandem repeats hosting the active cysteine residue in the C-terminus, as single domain proteins, and in combination with distinct proteins domains. The prototype for a single domain Str protein is found in *Escherichia coli* (GlpE) that often interacts with thioredoxins [4,5]. It has been reported that single rhodanese domain proteins are involved in reactions to stress defense, such as the *Drosophila melanogaster* heat shock protein 67B2, the *E. coli* phage shock protein PspE [6] or the *Vibrio cholerae* shock protein q9KN65 [7]. In plants, proteins with single rhodanese domains are associated with the process of leaf senescence, for example in *Arabidopsis thaliana*, *Nicotiana tabacum and* *Raphanus sativus* (Sen1, Ntdin and Din1, respectively) [8,9]. However, the mode of action in response to stress or senescence processes is not yet known. The proteins, composed of two rhodanese domains with the catalytic cysteine in the C-terminal rhodanese domain, are represented by the bovine mitochondrial rhodanese [10] and the *Azotobacter vinelandii* rhodanese (RhdA) [11]. The amino acid composition of the active site loop containing the active cysteine residue affects the substrate recognition and specificity [12]. Notably, changing of the active site loop by one additional amino acid influences the substrate specificity of *A. vinelandii* RhdA from sulfate- to phosphate-containing compounds [13,14]. In the *N*-terminal domain, the cysteine residue is often replaced by aspartic acid or glycine and found to be associated with other protein domains such as MAPK phosphatases [15]. Certain stress response proteins and several ubiquitinating enzymes are also proposed to share the non-catalytic rhodanese homology [14,16]. Therefore, it has been suggested that the inactive rhodanese domain could be involved in signaling [12] but more experimental evidence is needed.

Sulfurtransferases or Str-like proteins have been identified in different subcellular compartments. In rats, 3-mercaptopyruvate Str was identified in the cytoplasm and in mitochondria. It has been suggested to be involved in cyanide detoxification in the cytoplasm and to protect cytochrome c oxidase in mitochondria [17]. There are 20 different Strs or Str-like proteins in *A. thaliana* [18,19]. These have been classified into six groups based on their amino acid sequence similarities [20]. In wheat, Str was found to be involved in the resistance against the fungal pathogen *Erysiphe graminis* [21]. In another study, a cadmium-induced *A. thaliana* Str9 (AtStr9) homologue was identified in *Datura innoxia,* indicating a role of this Str in heavy metal stress [22]. Str/rhodanese domains are structurally similar to the catalytic subunit of arsenate reductase and Cdc25 phosphatase [14]. The high abundance of Str proteins in *A. thaliana* and other plant species [1] in different cellular compartments is speculated to pave the way for several specific biological functions, especially in abiotic and biotic stress defense.

## 2. Detoxification of Cyanide

Plants are exposed to cyanide from different exogenous and endogenous sources. The largest source of cyanide in the environment comes from anthropogenic activities, like soil contaminated by various industrial wastes containing up to 11,000 mg cyanide kg^−1^ DW soil [23]. Some natural exogenous sources including bacteria, fungi, algae, and neighboring plants are also responsible for cyanogenesis in significant amounts. The endogenous source of cyanide in plants is mainly the conversion of 1-amino-cyclopropane-1-carboxylic acid to ethylene that produces cyanide in equimolar amounts as ethylene and is drastically increased during fruit ripening and senescence [24]. Cyanide is also a potent inhibitor of respiration by inhibiting cytochrome c oxidase. Plants can readily take up cyanide and metallocyanides when present in the root zone [25]. Cyanide induces the formation of reactive oxygen species (ROS) and also triggers the production of hydrogen peroxide (H_2_O_2_) in embryonic axes of sunflower (*Helianthus annuus* L.) by stimulating NADPH oxidase and inhibiting antioxidant enzymes for instance catalase [26]. In higher plants, two metabolic pathways are involved in the detoxification and assimilation of excess cyanide. The first one is the Str pathway, also observed in bacteria and mammalians. In mammals, Strs play the crucial role in catalysis of cyanide and in the formation of the less toxic thiocyanate that is primarily excreted in the urine [27]. In *Pseudomonas aeruginosa,* mitochondrial rhodanese has been proved to be involved in the protection of aerobic respiration from cyanide poisoning by transferring sulfane sulfur from thiosulfate to cyanide and yielding less toxic thiocyanate [28]. In plants, the contribution of Str to cyanide detoxification may be negligible or incidental [29]. Most recently, it was observed that the β-cyano-l-alanine (β-CAS) pathway is the principal mechanism for maintaining cyanide homeostasis in higher plants [30]. Previously, it was already suggested that β-cyano-l-alanine synthase (CAS) plays a more important in cyanide detoxification than Str activity in *A. thaliana* [29]. At first, cyanide is substituted for the sulfhydryl group of cysteine to form β-CAS with the release of hydrogen sulfide [31]. Subsequently, the β-CAS is hydrolyzed by the gene product of NIT4, a dual enzyme with nitrilase and nitrile hydratase activity, yielding asparagine, aspartate and ammonia, respectively [30,32]. However, this study also explained, that the minor contribution of Str in cyanide detoxification could be based on methodological problems in the determination of volatile hydrogen cyanide and cyanogenic compounds in plant tissue. Future work is needed to finally clarify the role of Str in cyanide detoxification in different environmental and developmental conditions.

## 3. Detoxification of Reactive Oxygen Species

**Scheme 2:**
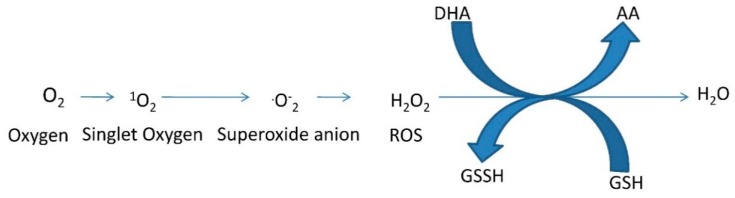
A flow chart of ROS formation and its detoxification. GSH (glutathione), GSSH (oxidized glutathione), AA (ascorbic acid) and DHA (dehydroascorbate) [35].

Different cellular compartments such as chloroplasts (photosystems I and II), mitochondria (complex I, ubiquinone, and complex III of the electron transport chain), and peroxisomes are the major sites of formation of ROS [33]. Despite of those compartments, heavy metal ions mediate reactions (e.g., Fenton reaction) that exert an effect on the production of ROS leading to a decreased level of available antioxidant reserves [23]. Reactions with ROS damage proteins, lipids, carbohydrates, and DNA, ultimately yield in oxidative stress. Against this backdrop, plants possess an antioxidant defensive machinery to protect against stress damage. The tripeptide glutathione is an important antioxidant in many organisms preventing damage to important cellular components caused by ROS such as free radicals and peroxides (Scheme 2) [34]. Glutathione in its reduced and oxidized forms, GSH and GSSG, plays a significant role within the cellular redox state by maintaining sulfhydryl (-SH) groups. Sulfurtransferases, for example thiosulfate-thiol Str, are enzymes that participate in GSH metabolism and homeostasis [34].

Various enzymes, such as superoxide dismutase, catalase, glutathione reductase, and glutathione *S*-transferase (GST) work in concert to control the oxidative damage by scavenging ROS [36]. Glutathione reductase found in prokaryotes and eukaryotes plays a pivotal role in the defense system against ROS, and it is localized predominantly in chloroplasts, but small amounts were found in mitochondria and the cytosol. Glutathione reductase is involved in the maintenance of the ascorbate-GSH cycle and NADPH-dependent reaction of disulphide bond recovery of GSSH by sustaining the reduced status of GSH [37]. Glutathione peroxidase provides an alternative means of detoxifying activated oxygen by using GSH to reduce hydrogen peroxide, which then yields GSSG [35]. It has been observed that glutathione reductase activities increase in the presence of cadmium in *A. thaliana*, *Vigna mungo*, *Triticum aestivum*, and *Brassica juncea*. In another study, transgenic *Nicotiana tabacum* with 30%–70% less glutathione reductase activity showed enhanced sensitivity to oxidative stress. Likewise, GSH concentrations were also elevated with heavy metal induced oxidative stress [38,39]. Plant GSTs have a crucial role to remove cytotoxic or genotoxic compounds. They have been found in maize, soybean, and A. thaliana. GSTs have been noticed to reduce peroxides by the assistance of GSH and yield scavengers of cytotoxic and genotoxic compounds [40].

Str might play a role in the control of redox homeostasis in the different subcellular compartments in a protein-protein interaction with thioredoxin. In this process, Str might act as a thioredoxin peroxidase with the intermediate formation of a sulfenate at the active-site cysteine as summarized in [1].

## 4. Detoxification of Heavy Metals

Lead, cadmium, and mercury are profoundly toxic to tissue, cells and cellular components. It is known that the sulfur-containing endogenous compounds play a pivotal role in various physiological processes in organisms, such as the stabilization of protein structure and regulation of enzymatic activity, in addition to their role in redox reactions as described above. Notably, Str (rhodanese, 3-mercaptopyruvate Str and γ-cystathionase) plays an important role in the metabolism of L-cysteine [41]. The catalytic activity of these enzymes are decreased via heavy metals binding with -SH groups of cystenine residues [42]. Consequently, changes in the level of sulfane sulfur-containing compounds, products of l-cysteine desulfuration and glutathione, are observed. An alteration in the activity of Str after exposure to lead, cadmium, and mercury was noticed in kidneys, liver, heart, brain, and skeletal muscle of Marsh frog [42].

**Figure 1:**
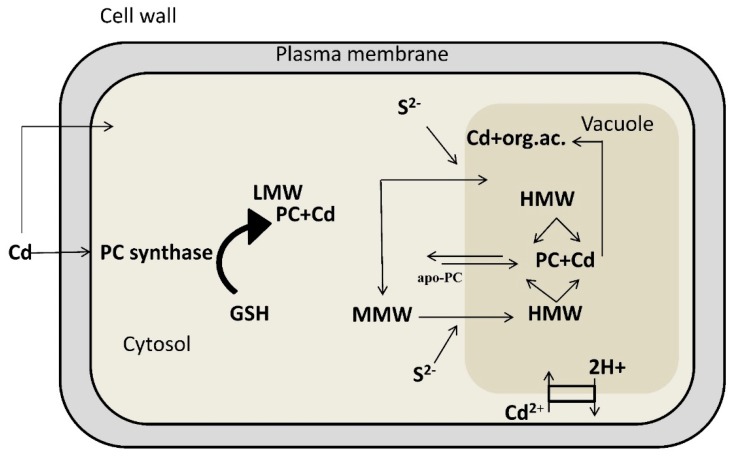
Diagram illustrating the mechanisms involved in cadmium chelation and compartmentalization in the vacuole (modified from ref. [43]). Phytochelatins are synthesized from GSH by the enzyme phytochelatin synthase. Exposure to cadmium stimulates synthesis of phytochelatins, which rapidly form a “low molecular weight” (LMW) complex with cadmium and a “medium molecular weight” (MMW) complex characterized by cadmium prevalently bound to phytochelatins with a higher polymerization level. At the tonoplast level, these complexes acquire acid-labile sulfur (S^2−^) and form a “high molecular weight” (HMW) complex with a higher affinity towards cadmium ions. Thus, particularly the HMW complex, highly stabilized by S^2−^ groups, seems to be decisive in cadmium detoxification. “LMW”, low molecular weight; “MMW”, medium molecular weight; “HMW”, high molecular weight; GSH, glutathione; PC, phytochelatins; apo-PC, apo-phytochelatins; S^2−^, acid-labile sulfur; org. ac., organic acids.

Abiotic stress factors, such as exposition to heavy metals, induce the expression of sulfate assimilation and sulfate transporter genes [44]. In plants, cysteine and GSH can be synthesized in all tissues but higher biosynthetic activities of enzymes involved in cysteine and GSH production were observed in *A. thaliana* trichomes, where presumably also phytochelatins are produced for heavy metal detoxification (Figure 1) [45].

To alleviate oxidative stress, GSH functions as a direct antioxidant and also as a reducing agent for other antioxidants such as ascorbic acid [35]. Cysteine is essential for GSH synthesis. Sulfur assimilation is also regulated by the cellular oxidative state. For example, an isoform of 5-adenylyl-sulfate (APS) reductase is activated by oxidation of two SH-groups of cysteine residues in the enzyme into a disulfide bond by oxidized glutathione [46]. It has been suggested that enzymes of sulfur metabolism and GSH synthesis are post-translationally modified and activated after consumption of reduced GSH by oxidative stress mitigation [47].

### 4.1. Sulfurtransferases with Arsenate Reductase Activity

Several plants species have been identified to accumulate arsenic in their plant tissues, for example the ferns *Pteris vittata* and *Pityrogamma calomelanos* [48]. The actual mechanisms of arsenic uptake and the manner in which plants detoxify these pollutants are not well known. Arsenate reductases (AR) are enzymes that catalyse the essential reduction reaction in the process of arsenic phytoremediation. Their active site contains a pair of cysteine residues that are essential for its catalytic action. One residue is part of the highly conserved sequence: Cys-(X)_5_-Arg. The mechanism of enzymatic reduction by AR involves formation of a thioester bond between the cysteine and As (V). The arginine residue assists in the stabilization of the intermediate [49].

Arsenate reductase activity was determined in an arsenate-hyperaccumulating fern. The reaction mechanism was very similar to the previously reported activity of Acr2p from yeast, using GSH as the electron donor. A T-DNA knockout mutant of *A. thaliana* with disruption in the homologous *Acr2* gene showed no AR activity [50]. Recently, it has been suggested that one member of the Str family also acts as AR in *A. thaliana*. According to the nomenclature by Bartels* et al.* [18], the orthologous protein in *A. thaliana* corresponds to AtStr5 (At5g03455), one of the 20 existing proteins containing a rhodanese domain grouped along with three other Str into “Group III”. It is predicted to be localized in the nucleus and contains a cysteine residue in the active centre [1]. The active site loop of AtStr5 has also a His-Cys-(X)_5_-Arg motif. Interestingly, the same protein was shown to act as Cdc25 dual-specificity tyrosine-phosphatase that is involved in the progression of the cell cycle by the removal of inhibitory phosphate residues from target cyclin-dependent kinases (CDKs) [20,51]. Notably, the His-Cys-(X)_5_-Arg motif coincides with the protein tyrosine phosphatase signature motif,, but the regulatory N-terminal domain is absent in AtStr5 unlike human Cdc25 [52]. Thus, the rhodanese domain with seven amino acid loop is able to bind to substrates containing phosphorous or in a similar way to arsenic, whereas the rhodanese-like domains with the six amino acid loop interact with substrates containing reactive sulfur or in some cases selenium [53].

Arsenate reductase from* Saccharomyces cerevisiae* Acr2p (or *Sc*Acr2p) and *Pteris vittata* (*Pv*ACR2) has been predicted to have three-dimensional structure related to members of rhodaneses/Cdc25 superfamily and share the Cdc25 active site motif His-Cys-(X)_5_-Arg. They do not exhibit significant phosphatase activity, although *Sc*Acr2p can be converted from reductase to a phosphatase by a small number of mutations [54,55,56]. In contrast, AtStr5 (also named as AtACR2), *Os*ACR2 from rice *Oryza sativa*, and *Lm*ACR2 from the parasitic protozoan *Leishmania major* showed both arsenate reductase and phosphatase activities [50,57,58]. In another study, AtStr5 over-expressing *A. thaliana* lines were found to resemble wild-type plants without any indication of over-proliferation or increased cell cycle rates [59]. In the same study, the *AtStr5* T-DNA insertion knockout mutants and AtStr5 over-expressing lines were tested for altered behavior after auxin and cytokinin treatment, but no altered hormone response was observed. This contradicted the role of the *A. thaliana* Cdc25 homolog, the AtStr5 protein, as a regulator of the cell cycle progression.

### 4.2. AtStr5 as Arsenate Reductase: Possible Role in Arsenic Phytoremediation

**Figure 2:**
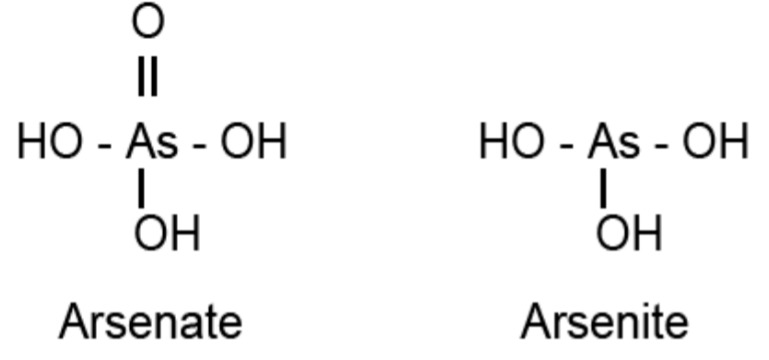
Inorganic forms of arsenic.

Arsenic occurs in the environment mainly in its inorganic form, as arsenite [As (III)] and arsenate [As (V)]. Figure 2 gives the structures of the main inorganic forms of arsenic, arsenate and arsenite.

Both these forms of arsenic are toxic to organisms. However, As (III) is considered to be more toxic than the As (V) form. Both forms interrupt biological functions in a different manner. Arsenite binds to proteins with sulfhydryl groups interfering with their functions. It inhibits respiration by binding to vicinal thiols in pyruvate dehydrogenase and 2-oxo-glutarate dehydrogenase [60]. Arsenite does not act directly as a mutagen but induces intra-chromosomal homologous recombination [61] and generates ROS [62], whereas As (V) interferes with oxidative phosphorylation and ATP synthesis [63].

**Figure 3:**
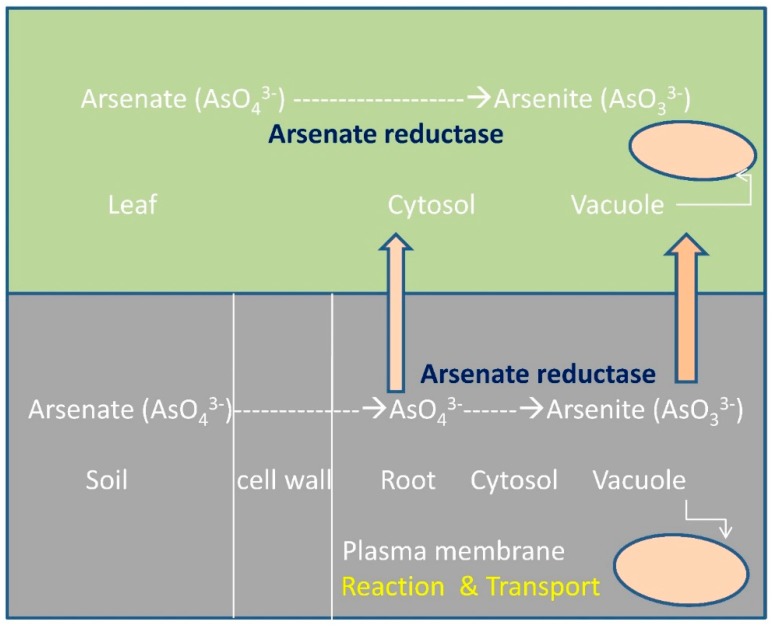
Mechanism of arsenic phytoremediation (modified from [25]).

The arsenic hyperaccumulator plants are potential candidates for arsenic phytoremediation. The process by which the plant accumulates arsenic is illustrated in Figure 3. The As (V) uptake occurs via phosphate transporters, whereas As (III) influx takes place in its neutral As(OH)_3_ form through aquaglyceroporins. The majority of arsenic found in the soil is in the arsenate form bound to different elements with different solubility, also dependent on the pH of the soil. The As (V) taken up is then reduced to As (III) by the enzyme AR. The next process is arsenite complexation with free thiol groups in order to detoxify the compound. This is followed by the vacuolar compartmentalization and storage of the arsenite-thiolate complex, thus completing the arsenic phytoremediation process [64].

The mechanism of enzymatic reduction by AR involves the formation of a thioester bond between the cysteine and As (V), and the arginine residue assists in the stabilization of the intermediate. The reduced form of AR is its active form, and the reducing agent involved in the generation of the active form of AR is either a thioredoxin or a glutaredoxin [65]. For example, AR encoded by *ArsC* gene from *Staphylococcus aureus* utilizes thioredoxin as its reducing agent, while the AR from *E. coli* R773 (encoded by *ArsC* gene) and *S. cerevisiae* (encoded by *Acr2p* gene) utilize glutathione and glutaredoxin as reducing agents [66].

So far the AR activity could not be confirmed for the recombinant AtStr5 *A. thaliana* protein. However, the comparison of growth of *A. thaliana* AtStr5-expressing *E. coli* cells and negative control cells cultured on media containing varying levels of arsenate (125 to 1000 µM) showed that AtStr5 positive *E. coli* transformants were resistant to arsenate (Papenbrock and co-workers, unpublished results). These observations further pointed towards the role of AtStr5 as an AR, maybe in an interaction process with thioredoxins.

In another study, the AR activity was observed in root extracts of T-DNA knockout *AtStr5* mutant plants unexposed to arsenate. Furthermore, it was confirmed that the AR activity of AtStr5 represents 36% of the total activity and is inducible by arsenate in *A. thaliana* roots [57]. In a similar study on plants grown under low arsenate exposure levels, the *AtStr5* mRNA was silenced using an RNAi construct. This increased the shoot arsenic accumulation 10–16 fold more than the roots of wild-type plants grown under identical conditions [59]. In contrast, the T-DNA insertion mutants of *AtStr5* accumulated less arsenic in shoots than wild-type plants over a range of arsenate concentrations [57]. Despite all these studies, the actual functional significance of AtStr5 is still a mystery. Discrepancy of these studies and broad physiological functions make AtStr5 a protein of further research interest. 

**Figure 4:**
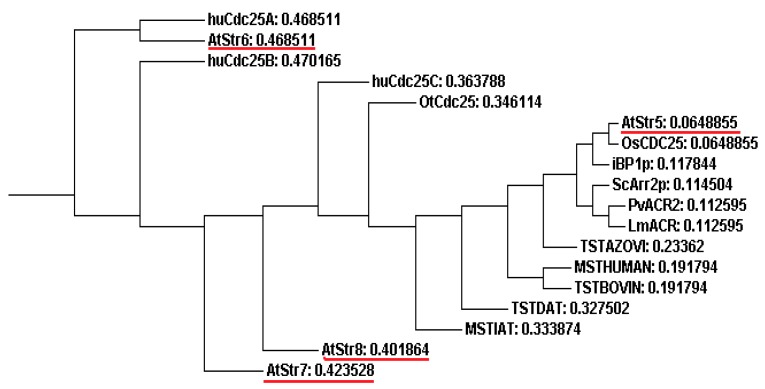
Phylogenetic tree obtained by using the Neighbor Joining method. The sequences selected for phylogenetic analysis are: Human Cdc25A (huCdc25A; NP_001780.2), Human Cdc25B (huCdc25B; NP_068658.1), Human Cdc25C (huCdc25C; NP_073720.1), *Ostreococcus tauri* Cdc25 (OtCdc25; AAQ16122.1), *Oryza sativa* Cdc25 (OsCdc5; AAX54896.1), Itsy bitsy phosphatase 1(iBP1p; Q8WZK3.1), *Saccharomyces cerevisiae* arsenate reductase (ScArr2p; NP_015526.1), *Pteris vittata* arsenate reductase (PvACR2; ABC26900.1), *Leishmania major* arsenate reductase (LmACR; AAS73185.1), *Arabidopsis thaliana* Group III sulfurtransferases (AtStr5; AAO39886.1, AtStr6; ABO38777.1, AtStr7; ABF57279.1, AtStr8; NP_564039.6), *Arabidopsis thaliana* 3-MP sulfurtransferase (MSTIAT; CAB64716.1), *Datisca glomerata* TS sulfurtransferase (TSTDAT; AAD19957.1), *Homo sapiens* 3-MP sulfurtransferase (MSTHUMAN; P25325.3), *Bovine taurus* TS sulfurtransferase (TSTBOVIN; P00586.3), *Azotobacter vinelandii* TS sulfurtransferase (TSTAZOVI; P52197.1). 3-MP, 3-mercaptopyruvate; TS, thiosulfate.

A distance phylogenetic tree (ClustalW tool from EBI based on Neighbor Joining) of rhodanese/Cdc25 superfamily members demonstrates the relationships among putative AR proteins (Figure 4). AtStr5 shows 55% identity and 79% similarity with *Oryza sativa* Cdc25 (OsCdc25), and 42% identity and 61% similarity with *Pteris vittata* AR. Although AtStr5 shares the same active site region as the human Cdc25 isoforms (A, B and C), it was seen that they have less overall identity. This may be considered as another indication for the possibility that AtStr5 may not act as a phosphatase but may have an important role in arsenate reduction. AtStr6 is similar to the human Cdc25 isoform A (huCdc25A) and since huCdc25A has no AR activity, the same can be expected of AtStr6. AtStr7 and AtStr8 form an entirely different clade in the phylogenetic tree, suggesting that they are not functionally related to AtStr5. Therefore, the other Str from group III, except AtStr5, may not show AR activity.

### 4.3. Phytoremediation

Phytoremediation is virtually considered as a potential solution to mitigate arsenic pollution. Certain plants known as hyperaccumulators or metallophytes have the ability to reduce heavy metal contamination by accumulating higher than normal levels of toxic heavy metals in their above-ground parts [64]. The over-expression of two bacterial proteins, AR encoded by the arsC gene and the γ-glutamylcysteine synthase, has been studied in A. thaliana in an attempt to yield a transgenic arsenic hyperaccumulator. Notably, AR catalyzes the reduction of arsenate to arsenite (AsO_3_^3−^) in the stem and leaves. The γ-ECS is involved in the first step of the phytochelatin synthesis pathway. γ-Glutamylcysteine complexes are formed with As (III) via its thiol groups, consequently detoxifying and preparing them for being stored away in vacuole, supporting the idea that the transgenic plants were able to accumulate 2–3 times more arsenic than wild-type plants [59]. However, the means by which arsenite-thiolate compounds are transported into the vacuole is still unknown.

## 5. Conclusions

Various heavy metal ions trigger the overproduction of ROS or free radicals in plants which are toxic and highly sensitive to proteins, lipids, carbohydrates and DNA, and ultimately results in oxidative stress. Against this backdrop, cells are evolved with sophisticated antioxidant defense mechanisms to detoxify the deleterious consequences of ROS. These antioxidant defenses could be non-enzymatic (e.g., glutathione, proline, carotenoids and flavonoids) or enzymatic (e.g., superoxide dismutase, GR and GSTs). There is profound evidence that cyanide leads to the yield of ROS and to escalate hydrogen peroxide (H_2_O_2_) by stimulating NADPH oxidase. In this context, β-CAS pathway has been profoundly accepted for maintaining cyanide homeostasis in higher plants. On the other hand, Str in animals has been well described as means of detoxifying cyanide. Nevertheless, Str activities are present in many living organisms, but their physiological role hare still ambiguously. Their ubiquity suggests additional physiological functions. Furthermore, it was suggested that one member of Str mimic as AR which is involved in arsenic phytoremediation and might be a promising candidate for successful removal of arsenic from soil. Henceforth, development of abiotic stress-tolerant crop via over-expression of ROS-scavenging enzymes and enzymes containing reactive sulfur groups, such as Str, may be useful.
